# THE BRIDGE CARE MODEL: TRANSITIONAL CARE FOR PERSONS EXPERIENCING HOUSELESSNESS

**DOI:** 10.1093/geroni/igae098.0512

**Published:** 2024-12-31

**Authors:** Heather Leutwyler, Taylor Cuffaro

**Affiliations:** University of California San Francisco, Pacifica, California, United States; University of California San Francisco, San Francisco, California, United States

## Abstract

**Introduction:**

Persons experiencing houselessness (PEH) are well-understood to prematurely age, experiencing higher rates of geriatric conditions at a younger age, considered “older” by age 50. Based on the rigorously-tested Transitional Care Model for older adults, UCSF Street Nursing provides low barrier care to PEH during transitions from emergency departments (ED) and in-patient admissions. Our practice focuses on: nurse-led early assessment and engagement; continuity and coordination of care; the patient-clinician relationship; holistic focus on individual patient’s needs; education and promotion of self-management; active engagement of patients and their support network; and patient-driven evidence-based care planning to address health problems and risk factors that contribute to frequent ED visits and hospital readmissions among PEH.

**Methods:**

UCSF Street Nursing meets patients in the ED or in-patient prior to discharge and follows them for 6-12 months, supporting integration into community outpatient care. Clinical activities include: medication administration, wound care, street-based syphilis and STI treatment, complex care coordination, complex medication management, health education focused on self-management, and co-attending primary and specialty care visits both virtually and in-person. Outcomes: The average age of the 58 patients referred to our practice was 52 (sd=12.5). Given the premature mortality and accelerated aging of PEH, our sample represents an older adult population. Our practice produced a greater than 50% reduction in ED visits in the first 6 months following discharge.

**Conclusion:**

UCSF Street Nursing is a promising nurse-led novel approach to care for unhoused older adults with potential to reduce hospitalization and improve health for an overlooked population.

